# Differences in functional traits of herbaceous plants in Sanjiang plain wetland under human disturbance gradient and their response strategies to environmental changes

**DOI:** 10.3389/fpls.2025.1733287

**Published:** 2026-01-22

**Authors:** Qiuyu Meng, Zihe Liu, Naixu Guo, Jiping Liu

**Affiliations:** 1 College of Geographic Science and Tourism, Jilin Normal University, Siping, China; 2 College of Life Sciences, Jilin Normal University, Siping, China

**Keywords:** environmental factors, human interference gradient, plant functional traits, response strategy, Sanjiang Plain

## Abstract

Using the Honghe Nature Reserve in the Sanjiang Plain as a case study, this research tests the central hypothesis that increasing anthropogenic disturbance intensity shifts the ecological strategies of dominant plant species along a resource-conservative to acquisitive spectrum, thereby progressively enhancing the role of soil properties as proximate drivers of trait variation. This hypothesized process unfolds sequentially: under low-intensity disturbance, direct physical stress acts as the primary filter on traits; at moderate intensity, disturbance begins altering soil conditions, shifting plant adaptation toward soil resource competition and increasing the explanatory power of soil factors; under high-intensity disturbance, profoundly transformed soil environments become the dominant proximate filter, selecting strongly for resource-acquisitive traits. We examined this framework by measuring leaf and root traits of 14 dominant herbaceous species and soil factors across 24 disturbance-gradient plots. Findings confirm distinct adaptive strategies: phosphorus-limited growth in light disturbance, conservative resource use in moderate disturbance, and a shift toward fast-return strategies in fertile, heavily disturbed soils. This study mechanistically traces the cascade from ultimate (anthropogenic disturbance) to proximate (soil) drivers of plant adaptation, providing a scientific basis for targeted wetland restoration that addresses disturbance legacies.

## 1 Introduction

Wetland ecosystems account for approximately 5-8% of the global land area and play important roles in climate regulation, flood storage, nutrient cycling and biodiversity maintenance. However, due to human activities such as agricultural expansion, industrial pollution and urbanization, the area of wetlands has continued to decrease and their ecological functions have significantly deteriorated (Tang, 2022). As a key component of the wetland ecosystem, the functional traits of plants reflect the adaptation strategies to environmental changes and regulate key ecological processes such as nutrient cycling and carbon fixation (Violle et al., 2007; Li et al., 2024). Therefore, studying the response mechanisms of plant functional traits to environmental factors is of great significance for predicting ecosystem dynamics and guiding protection and restoration.

Plant functional traits are closely related to soil characteristics. Factors such as soil moisture, nutrients, salinity and REDOX potential regulate the acquisition and distribution strategies of plant resources (Bernhardt-Römermann et al., 2011). Facing the gradient changes of resource availability and environmental pressure, plants adjust resource allocation through trade-off mechanisms (Chen and Xu, 2014). For example, in seasonally flooded wetlands, soil hypoxia promotes the development of aeration tissues in plants, accompanied by a lower root length and a higher root tissue density to enhance hypoxia tolerance (Lü et al., 2010), reflecting the trade-off between oxygen acquisition and structural support (Cheng et al., 2022). Nutrient availability drives leaf traits to differentiate along the “conservation-acquisition” spectrum: high nutrients promote high specific leaf area and high leaf nitrogen content, supporting rapid growth; Poor soil screened out conserved species with high leaf dry matter content and low specific leaf area (Wright et al., 2004), reflecting the trade-off between growth rate and resource conservation.

Human interference has become the key driving force for reshaping the “soil-plant” relationship (Chu et al., 2023). Activities such as grazing, infrastructure construction, and drainage indirectly affect plant functional traits by altering the physical and chemical properties of the soil, compelling plants to make new trade-offs in resource allocation (Diaz et al., 2004). Grazing activities screened out plants with tolerance traits such as low growth point, high tissue density and high root biomass investment (Gao et al., 2021); Infrastructure construction has led to habitat fragmentation and pollution, prompting plants to develop higher heavy metal tolerance, lower specific leaf area and deeper root systems (Haber et al., 2023); Drainage activities lower the groundwater level, promote the decomposition of organic matter and nutrient mineralization, and drive the succession of plant communities to rapid resource acquisition type (Heberling and Fridley, 2012). These studies show that plants optimize resource allocation through the adjustment of trait combinations and form adaptive strategies for different levels of disturbance intensity.

The theories of “leaf economic spectrum” and “root economic spectrum” provide a systematic framework for understanding the response of plant traits (Wright et al., 2004; Roumet et al., 2016), respectively depicting the trade-off continuum between resource acquisition and preservation of leaves and roots. Wetland plants exhibit unique forms of trait trade-off expression when confronted with hydrological fluctuations and human disturbances (Hu et al., 2024). However, the existing research still has deficiencies: most focus is on leaf traits or a single type of interference, lacking systematic observations of the coordinated response of above-ground and underground traits to compound disturbances (Huang and Wang, 2003); Less attention has been paid to the mediating role of soil in the interference-trait relationship, and the “interference intensity - soil change - trait response” pathway and the resource trade-off mechanism behind it have not been fully revealed (Ji et al., 2020).

The Sanjiang Plain, as the largest freshwater marsh wetland in China, holds significant ecological functions and conservation value (Jiang et al., 2006). This area is confronted with multiple human pressures such as agricultural reclamation, water resource development, infrastructure construction, and grazing. As a result, the area of natural wetlands has significantly decreased, and the habitat has become severely fragmented and degraded, forming a continuous gradient of disturbances ranging from mild to severe (Ke et al., 2014). This gradient provides an ideal experimental field for studying the adaptation strategies of plants under different types and intensities of interference (Kong et al., 2017). Therefore, this study takes the Honghe Nature Reserve in the Sanjiang Plain as the research object. By measuring the functional traits of leaves and roots of dominant herbaceous plants and soil factors under different interference gradients, it aims to reveal: (1) the variation law of functional traits of wetland plants along the interference gradient; (2) The mediating role of soil factors; (3) Adaptation strategies and resource trade-off mechanisms of plants under different interference intensifies. The research results can provide a scientific basis for wetland protection and restoration.

## 2 Materials and methods

### 2.1 Research area and species

**Figure 1:**
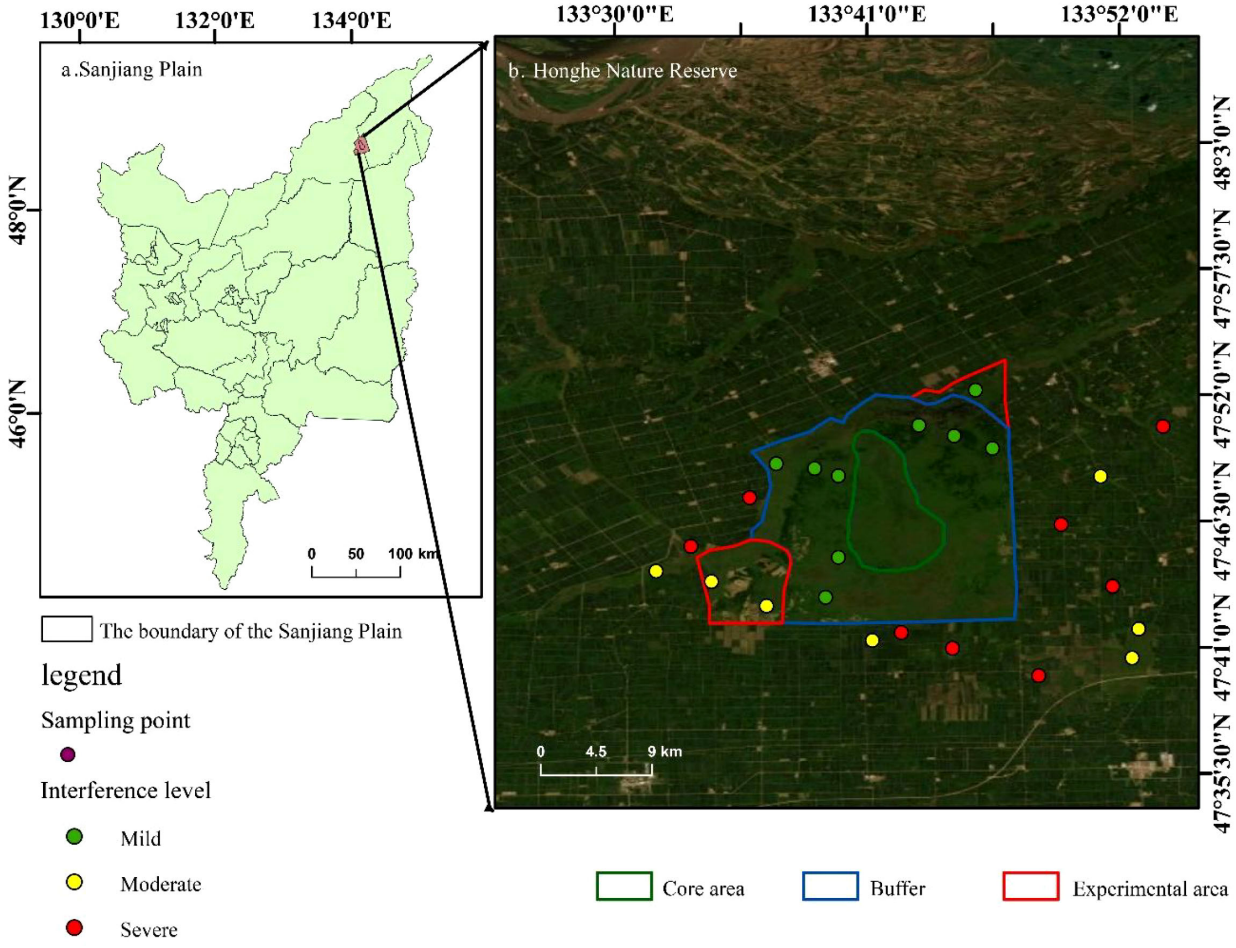
Geographical location of study area.

The study was conducted in the Sanjiang Plain, located in northeastern Heilongjiang Province, China. This region represents one of China’s largest concentrated distribution areas of freshwater wetlands, with a total area of 10.89×10^4^ km² (Wang et al., 2013). Our research focused specifically on the Honghe National Nature Reserve (133°34′38″E~133°46′29″E, 47°42′18″N~47°52′07″N) and its surrounding areas, covering approximately 21,836 hectares of pristine freshwater swamp wetland (**Figure 1**). The typical soil conditions in this area are mainly marsh soil and meadow soil. These soils generally have a high organic matter content and water-holding capacity, but their physical and chemical properties are significantly affected by hydrological changes and human activities (Li et al., 2018).

The range of human disturbance in the study area covers from the strictly protected core wetlands to the edge areas with dense human activities, forming a complete gradient of disturbance intensity (Li et al., 2019). Specific types of disturbances include light to moderate grazing, medium to high-intensity agricultural drainage and reclamation, as well as habitat edge effects caused by road construction. This clear gradient provides an ideal natural experimental field for systematically examining the response mechanism of plant functional traits to differentiated anthropogenic stress (Liu, 2012).

The main plant species in the area are herbaceous plants adapted to the wetland environment. The dominant species include *Sedge* and *false sedge* of the Cyperaceae family, as well as *reed* and *purple grass* of the Poaceae family (Lu and Zhang, 2014). This study systematically selected 14 dominant herbaceous plants as the analysis objects, which belong to 8 families and 12 genera, representing different ecological adaptations from hygrophytes to mesophytes, and can well reflect the functional and structural characteristics of the wetland plant community in this area.

In order to accurately test whether there is spatial autocorrelation in the sample plot, we use the classical indicator of Moran’s index. The value range of Moran’s index is between -1 and 1, which can intuitively reflect the nature and degree of spatial autocorrelation (Luke McCormack et al., 2012). Constructing an appropriate spatial weight matrix is a crucial step in the calculation process. We use distance criteria to determine the spatial weight matrix based on the actual geographical location information of the sample site (Ma et al., 2024). Set a reasonable distance threshold. When the distance between two plots is less than this threshold, their corresponding elements in the spatial weight matrix are denoted as 1, indicating that these two plots are spatially correlated; Otherwise, it is recorded as 0.

By calculating the Moran’s index based on plant functional trait data and relevant soil factor data from 24 plots, and conducting significance tests, we found significant spatial autocorrelation (Mou et al., 2018). This result implies that the plant functional traits or soil factors between adjacent plots are not independent of each other, but rather have a certain degree of similarity or correlation.

Given the significant spatial autocorrelation, it must be fully considered in the subsequent construction of statistical models. We choose to incorporate spatial coordinates into the statistical model and use a spatial lag model for analysis. This model introduces a spatial lag term for the dependent variable, fully considering the influence of adjacent plots on the observed values of the current plot. For example, when analyzing the relationship between plant leaf and root traits and soil factors under different artificial disturbance gradients, spatial lag models can more accurately analyze the actual effects of each factor.

### 2.2 Data collection

In this study, to ensure the scientific validity of the analysis results, we conducted comprehensive and detailed normality and homogeneity of variance tests on all measurement data (Nosetto et al., 2024).

In terms of normality testing, we used the Shapiro Wilk Test. This testing method has high testing efficiency for small sample data and can accurately determine whether the data conforms to the assumption of normal distribution. We strictly follow the testing steps and input the data into professional statistical software to obtain the Shapiro Wilk test statistic and corresponding P-value for each dataset. If the P-value is greater than the pre-set significance level (usually 0.05), the data is considered to follow a normal distribution; On the contrary, it is determined that the data does not follow a normal distribution.

We used Levene’s test for homogeneity of variance. This testing method has relatively loose requirements for the distribution of data and can effectively evaluate the homogeneity of variance between different groups of data. By calculating the Levin test statistic and corresponding P-value, when the P-value is greater than the significance level, it indicates that the variance of each group of data is homogeneous; If the P-value is less than the significance level, it indicates uneven variance (O’Neill et al., 2013).

We did not directly discard data that did not meet the assumptions of normal distribution or homogeneity of variance after testing. Instead, we made appropriate transformation attempts, such as logarithmic transformation, square root transformation, etc., to make them meet the prerequisites for subsequent analysis as much as possible (Qu et al., 2018). If the conversion still cannot meet the requirements, we will use the Kruskal Wallis test in non parametric testing methods for subsequent analysis to ensure that scientific and reasonable research conclusions can be drawn under various data characteristics.

The field investigation was conducted in August 2023, and a total of 24 sample plots were set up along the human-induced disturbance gradient in the Honghe National Nature Reserve. The intensity of interference is classified into three levels - mild, moderate and severe - based on a comprehensive assessment of three quantifiable dimensions: road distance, grazing evidence and farmland proximity (Shi, 2021). Specifically, the severely disturbed plots are adjacent to roads or the edges of farmlands, with a distance from the roads not exceeding 100 meters, and are accompanied by obvious evidence of grazing activities, such as the amount of livestock manure per unit area exceeding 5 per 100 square meters, or significant reduction in vegetation height and obvious trampling marks on the soil surface. Moderately disturbed plots are 100 to 500 meters away from roads and are indirectly affected by surrounding farmlands or show signs of mild grazing. The amount of feces in these plots is generally 1 to 5 per 100 square meters. The mild disturbance sample is located at the core of the buffer zone, far from roads and farmlands. The distance from the road is more than 500 meters, and there are almost no traces of grazing activities. The amount of feces does not exceed 1 per 100 square meters or is completely absent (Song et al., 2023).

Set up a 10-meter by 10-meter large plot at the center of each plot, and arrange three 1-meter by 1-meter small plots along the diagonal for detailed vegetation investigation (Tang et al., 2024). Collect complete plants of dominant herbaceous plants such as purple-flowered wild grass, Carex, and reed. Meanwhile, soil samples were collected using a soil drill, sealed for storage and transported back to the laboratory for analysis. The coordinates of the sample plots were recorded using handheld GPS, and the soil temperature was measured *in situ* using a ground temperature meter.

The research area of this study has been scientifically planned and reasonably divided into 24 disturbance gradient plots. The division of these 24 plots is not arbitrary, but takes into account various environmental factors and differences in disturbance levels, aiming to comprehensively cover the ecological conditions under different levels of disturbance and provide a rich and representative sample basis for subsequent research (Thapa et al., 2022).

Within each carefully divided plot, we further set up three sampling points. The selection of these sampling points follows the principle of random and uniform distribution, striving to accurately reflect the overall ecological characteristics of the plot and avoid data distortion caused by the concentration or deviation of sampling points.

For each sampling point, we divided it into four quadrants and conducted measurements of plant functional characteristics for each quadrant. This detailed division method enables us to gain a deeper understanding of the various functional characteristics of plants from multiple perspectives and levels, and obtain more precise and accurate data (Wang et al., 2009).

Through this hierarchical sampling design, we ultimately calculate the total number of samples. The specific calculation method is: multiply 24 plots by 3 sampling points of each plot, and then multiply by 4 quadrants of each sampling point, that is, 24 × 3 × 4 = 288 samples.

### 2.3 Trait measurement

Specific Leaf Area (SLA), as a key indicator for measuring the functional characteristics of plant leaves, reflects the area size corresponding to the unit dry weight of leaves (Wang et al., 2019). It can comprehensively reflect the characteristics of plants in resource acquisition, utilization strategies, and growth adaptation. The calculation method strictly follows the standard protocol in the Functional Ecology Handbook, and the specific steps are as follows:

Firstly, collect the leaves. Randomly select several healthy, mature, and representative leaves within each selected sample quadrant. To ensure the accuracy and representativeness of the data, the sampling should cover leaves from different growth positions and lighting conditions.After the collection is completed, immediately use a precise area measurement tool, such as a leaf area meter, to measure the total area of the collected leaves (in square centimeters). The measurement process should ensure that the blades are placed flat to avoid measurement errors caused by folding, curling, and other factors.Subsequently, place the measured area of the blades into an oven and dry them to a constant weight at a suitable temperature (usually 70-80 °C). The drying time depends on the thickness and moisture content of the leaves, and usually takes several hours to tens of hours. After drying, use a precision balance to weigh the dry weight of the blades in grams.Finally, according to the definition of specific leaf area, the specific leaf area is calculated by dividing the total leaf area by the dry weight of the leaf. The formula is: SLA (square centimeters/gram)=total leaf area (square centimeters) ÷ dry weight of the leaf (grams).

In each quadrat, 3–5 intact, healthy individuals of dominant species were collected, ensuring the preservation of the root-shoot connection. Samples were immediately refrigerated (<5 °C) and transported to the laboratory for processing. Leaves and roots were scanned and analyzed using ImageJ and WinRHIZO Pro 2012b software, respectively (Wang et al., 2024).

The following functional traits were measured: Leaf Area (LA), saturated leaf fresh weight, leaf dry weight (after 48 h at 65 °C), Specific Leaf Area (SLA, leaf area per unit leaf dry mass), and Leaf Dry Matter Content (LDMC, leaf dry mass per unit saturated fresh mass). Leaf total carbon (LCC), nitrogen (LNC), phosphorus (LPC), and potassium (LKC) contents were determined using a total organic carbon analyzer and an elemental analyzer after ball milling.

Root traits included root length, volume, surface area, root dry weight (after 48 h at 65 °C), Specific Root Length (SRL, root length per unit root dry mass), Root Tissue Density (RTD, root dry mass per unit root volume), and Specific Root Area (SRA, root surface area per unit root dry mass). Root total carbon (RCC), nitrogen (RNC), phosphorus (RPC), and potassium (RKC) contents were analyzed using the same methods as for leaves (Wu et al., 2010).

### 2.4 Environmental data extraction

Soil moisture (SW, soil moisture,%), soil temperature (ST, soil temperature, °C), soil electrical conductivity (EC, soil conductivity, μS/cm), and soil salinity (SS, soil salinity, mg/L) were measured by using soil multi-parameter rapid detector. After natural air-drying, the soil samples were dried in an oven at 105 °C for 24 hours, ground and homogenized by a ball mill, and then passed through a 100-mesh sieve to determine the nutrient content. The total organic carbon content (SC, soil carbon concentration, mg/g) in soil samples was measured by Vario TOC, Elementar, Germany. The contents of total nitrogen (SN, Soil nitrogen concentration, mg/g) and total phosphorus (SP, Soil phosphorus concentration, mg/g) in soil samples were determined by an element analyzer (Vario EL III, Elementar, Germany).

### 2.5 Data analysis

All data were analyzed and charted by Origin 2021, SPSS 25.0, Canoco5, and R language 4.1. 3. Each trait variable was averaged at the quadrat level, and the variation degree of morphological traits and stoichiometric traits was calculated by using the coefficient of variation (CV). Before the analysis, the logarithmic transformation based on 10 was carried out to make the data satisfy the normalized normal distribution. Pearson correlation analysis was used to analyze the correlation between functional traits and environmental factors in different parts of plants. One-way ANOVA was used to test the difference in functional traits of leaves and roots under different human disturbance environments. Redundancy analysis (RDA) was used to explore the relationship between plant leaf and root traits and soil factors under different human disturbance types, to analyze the response mechanism of plants to different human disturbance environments.

## 3 Results and analysis

### 3.1 Variations in plant functional traits across disturbance gradients

#### 3.1.1 Leaf functional traits

**Figure 2:**
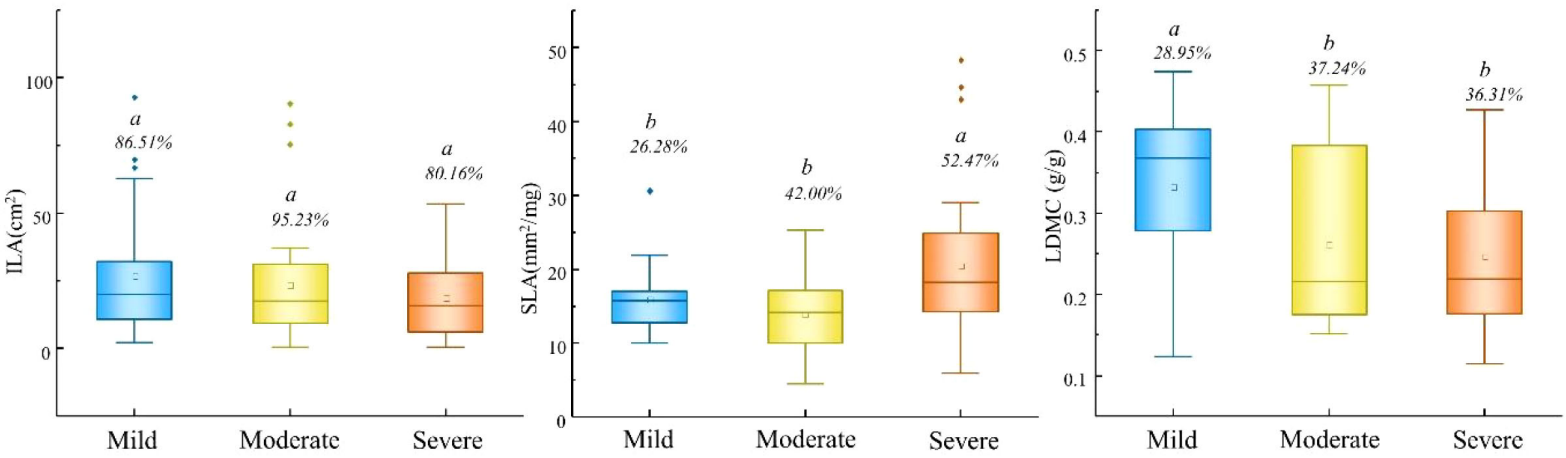
Differences in leaf morphological traits under different human disturbance gradients. ILA, leaf area; SLA, specific leaf area; LDMC, Leaf dry matter content.

Significant differences in leaf functional traits were observed across the human disturbance gradient (**Figure 2**). LA and LDMC were highest under mild disturbance, while SLA was significantly elevated under severe disturbance (*P* < 0.05). Specifically, SLA reached 20.51 mm²/mg under severe disturbance, significantly exceeding values under mild (15.91 mm²/mg) and moderate disturbance (14.00 mm²/mg). LDMC was significantly higher under mild disturbance (0.33 g/g) compared to moderate (0.26 g/g) and severe disturbance (0.25 g/g).

**Figure 3:**
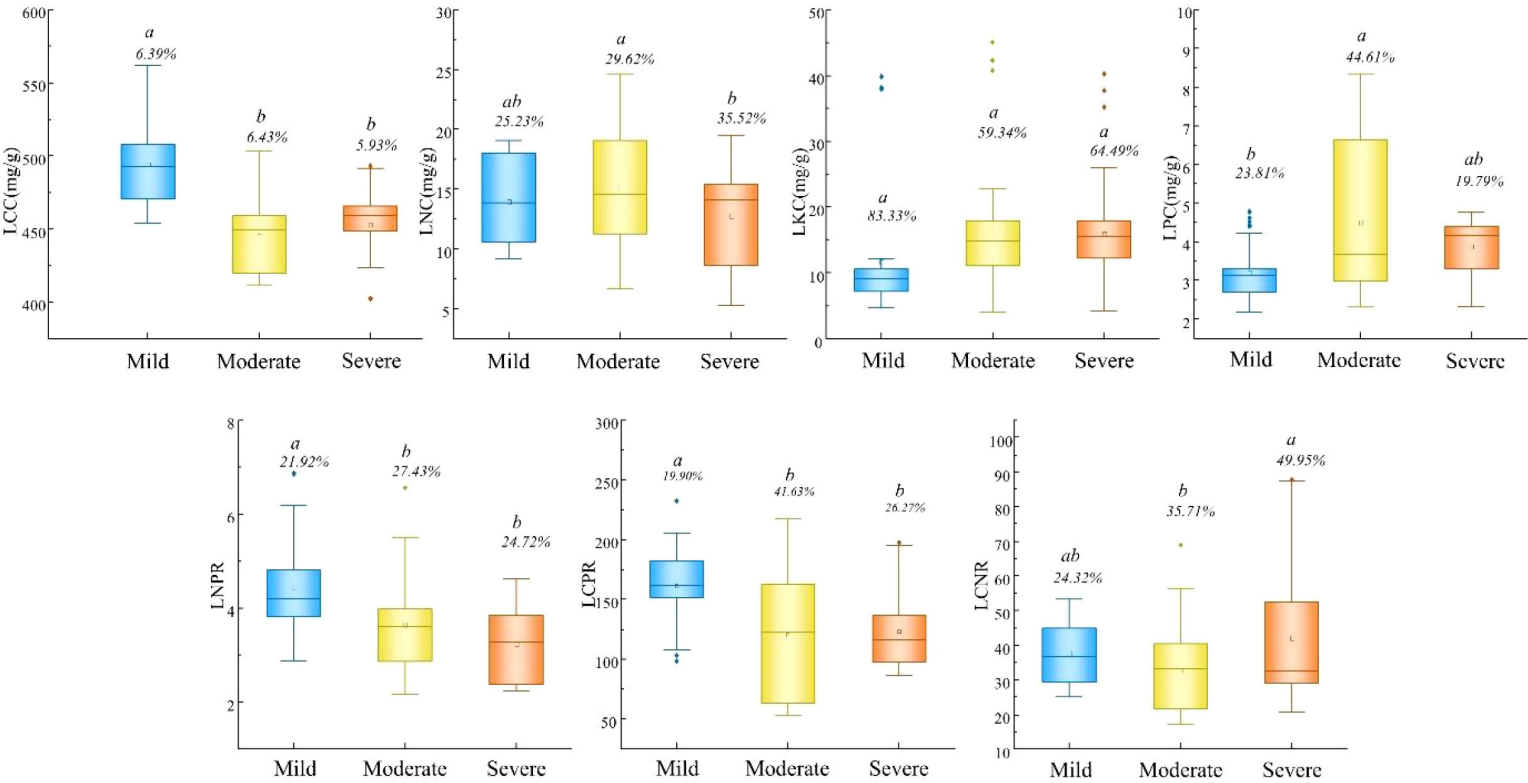
Difference of stoichiometric characteristics of leaves under different human disturbance gradients. LCC, leaf carbon content; LNC, leaf nitrogen content; LKC, leaf potassium content; LPC, leaf phosphorus content; LNPR, leaf nitrogen-phosphorus ratio; LCPR, leaf carbon-phosphorus ratio; LCNR, leaf carbon-nitrogen ratio.

Leaf stoichiometric characteristics also varied significantly with disturbance intensity (**Figure 3**). LCC was highest under mild disturbance (494.11 mg/g), significantly exceeding levels under moderate and severe disturbance. LNC peaked under moderate disturbance (15.14 mg/g), while LPC was also highest at this disturbance level (4.50 mg/g). LNPR and LCPR were significantly elevated under mild disturbance, whereas LCNR reached its maximum under severe disturbance.

#### 3.1.2 Root functional traits

**Figure 4:**
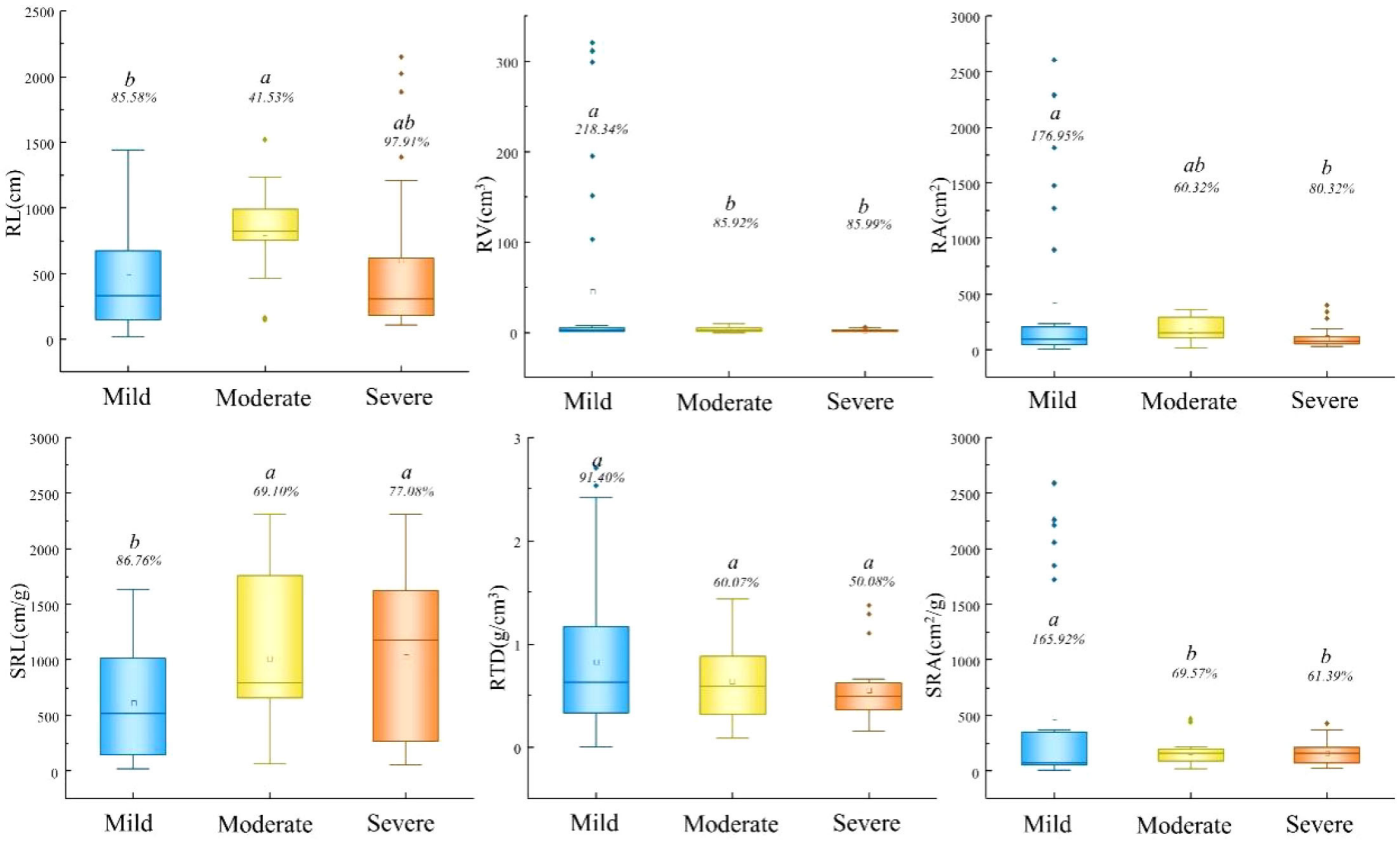
Differences of root morphological characters under different interference gradients. RL, root length; RV, root volume; RA, root surface area; SRL, specific root length; RTD, root tissue density; SRA, specific root area.

Root morphological traits demonstrated distinct patterns across disturbance gradients (**Figure 4**). RL was significantly longer under moderate disturbance (814.88 cm) compared to mild disturbance (471.98 cm). Conversely, RV and RA were substantially greater under mild disturbance (44.81 cm³ and 396.10 cm², respectively). SRL was significantly higher under both moderate (1016.07 cm/g) and severe disturbance (1031.49 cm/g) compared to mild disturbance, while SRA showed the opposite pattern.

**Figure 5:**
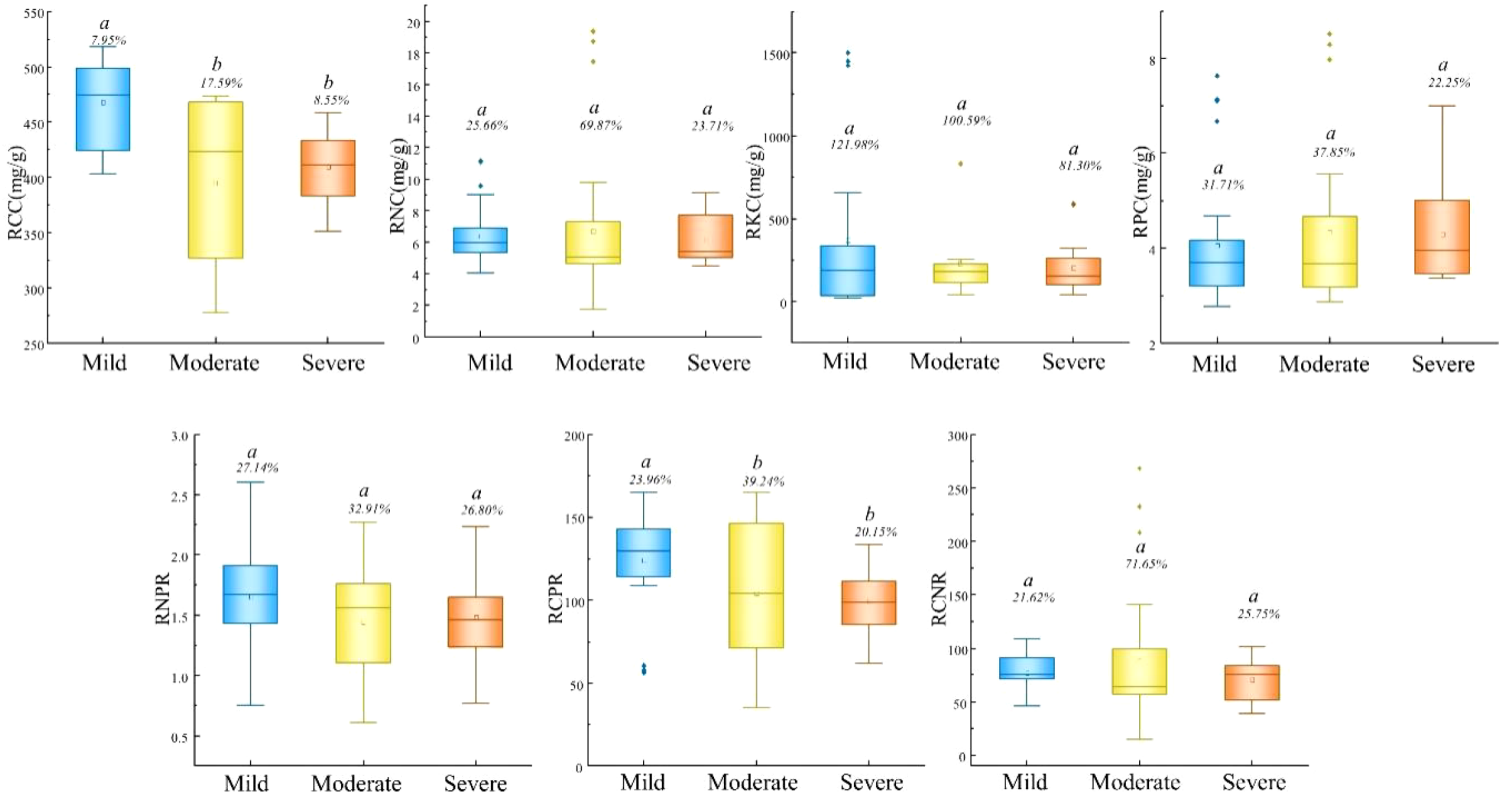
Difference of root stoichiometric characteristics under different interference gradients. RCC, root carbon content; RNC, root nitrogen content; RKC, root potassium content; RPC, root phosphorus content; RNPR, root nitrogen-phosphorus ratio; RCPR, root carbon-phosphorus ratio; RCNR, root carbon-nitrogen ratio.

Root stoichiometric traits exhibited less variation than morphological traits (**Figure 5**). RCC was significantly higher under mild disturbance (467.55 mg/g), but RNC, RPC, and RKC showed no significant differences across disturbance levels. Similarly, RNPR and RCNR remained stable, while RCPR was significantly elevated under mild disturbance.

### 3.2 Response of plant leaf and root traits to environmental factors

#### 3.2.1 Correlation with soil physical factors

**Figure 6:**
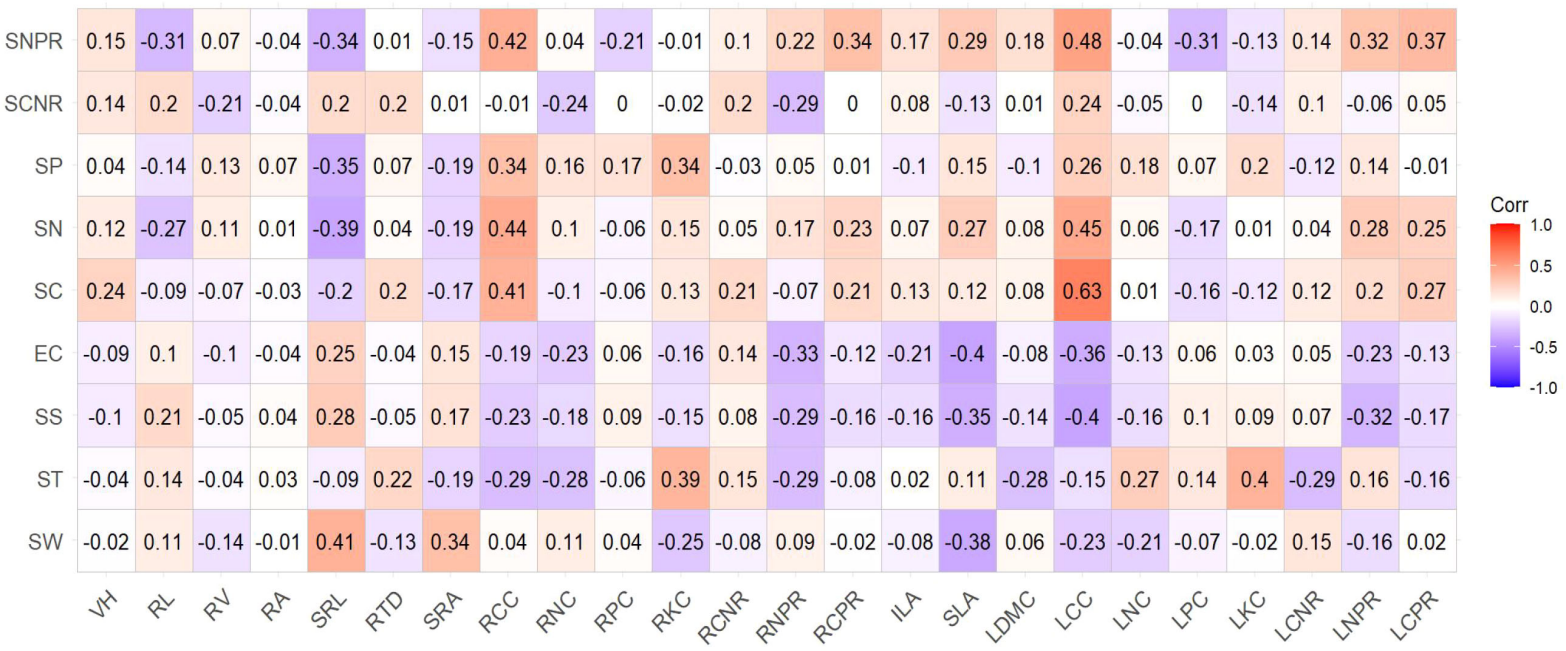
Response of plant functional traits to environmental factors. SW, soil moisture; ST, soil temperature; SS, soil salinity; EC, soil conductivity; SN, soil total nitrogen; SP, soil total phosphorus; SC, total soil organic carbon; SCPR, soil carbon-phosphorus ratio; SCNR, soil carbon-nitrogen ratio; ILA, leaf area; SLA, specific leaf area; LDMC, leaf dry matter content; LCC, leaf carbon content; LNC, leaf nitrogen content; LPC, leaf phosphorus content; LKC, leaf potassium content; LCNR, leaf carbon-nitrogen ratio; LCPR, leaf carbon-phosphorus ratio; LNPR, leaf nitrogen-phosphorus ratio; RL, root length; RV, root volume; RA, root surface area; SRL, specific root length; RTD, root tissue density; SRA, specific root area; RCC, root carbon content; RNC, root nitrogen content; RPC, root phosphorus content; RKC, root potassium content; RCNR, root carbon-nitrogen ratio; RCPR, root carbon-phosphorus ratio; RNPR, root nitrogen-phosphorus ratio. *P* = 0. 05 is a significant level, red indicates a significant positive correlation, blue indicates a significant negative correlation, and numbers indicate a correlation coefficient. *P* = 0. 05 significant level, significant positive correlations are indicated in red, significant negative correlations are indicated in blue, and numbers indicate correlations coefficients.

Pearson correlation analysis revealed significant relationships between plant functional traits and soil physical factors (**Figure 6**). SW showed strong positive correlations with SRL and SRA (*P* < 0.01), and negative correlations with RKC, LCC, and SLA (*P* < 0.05). ST was negatively correlated with RCC, RNC, RNPR, LDMC, and LCNR (*P* < 0.01), while positively correlated with RTD, RKC, LNC, and LKC (*P* < 0.05). Both SS and EC were positively correlated with SRL (*P* < 0.05) and negatively correlated with RNPR, SLA, LCC, and LNPR (*P* < 0.05).

#### 3.2.2 Correlation with soil nutrients

Soil nutrient factors demonstrated distinct relationships with plant traits (**Figure 6**). SC showed significant positive correlations with RCC and LCC (*P* < 0.01), as well as with RCNR, RCPR, and LCPR (*P* < 0.05). SN was positively correlated with RCC, LCC, and LNPR (*P* < 0.01), and with RCPR, LCPR, and SLA (*P* < 0.05), but negatively correlated with SRL (*P* < 0.01). SP exhibited significant positive correlations with RCC and RKC (*P* < 0.01), positive correlation with LCC (*P* < 0.05), and negative correlation with SRL (*P* < 0.01).

#### 3.2.3 Correlation with soil stoichiometric ratios

Analysis of soil stoichiometric ratios (**Figure 6**) indicated that SCNR was negatively correlated with RNC and RNPR (*P* < 0.05) and positively correlated with LCC (*P* < 0.05). SNPR showed negative correlations with RL, SRL, LPC, and RPC (*P* < 0.01), and positive correlations with RCC, RCPR, SLA, LCC, LNPR, RNPR, and LCPR (*P* < 0.01).

### 3.3 Adaptation strategies across disturbance gradients

**Figure 7:**
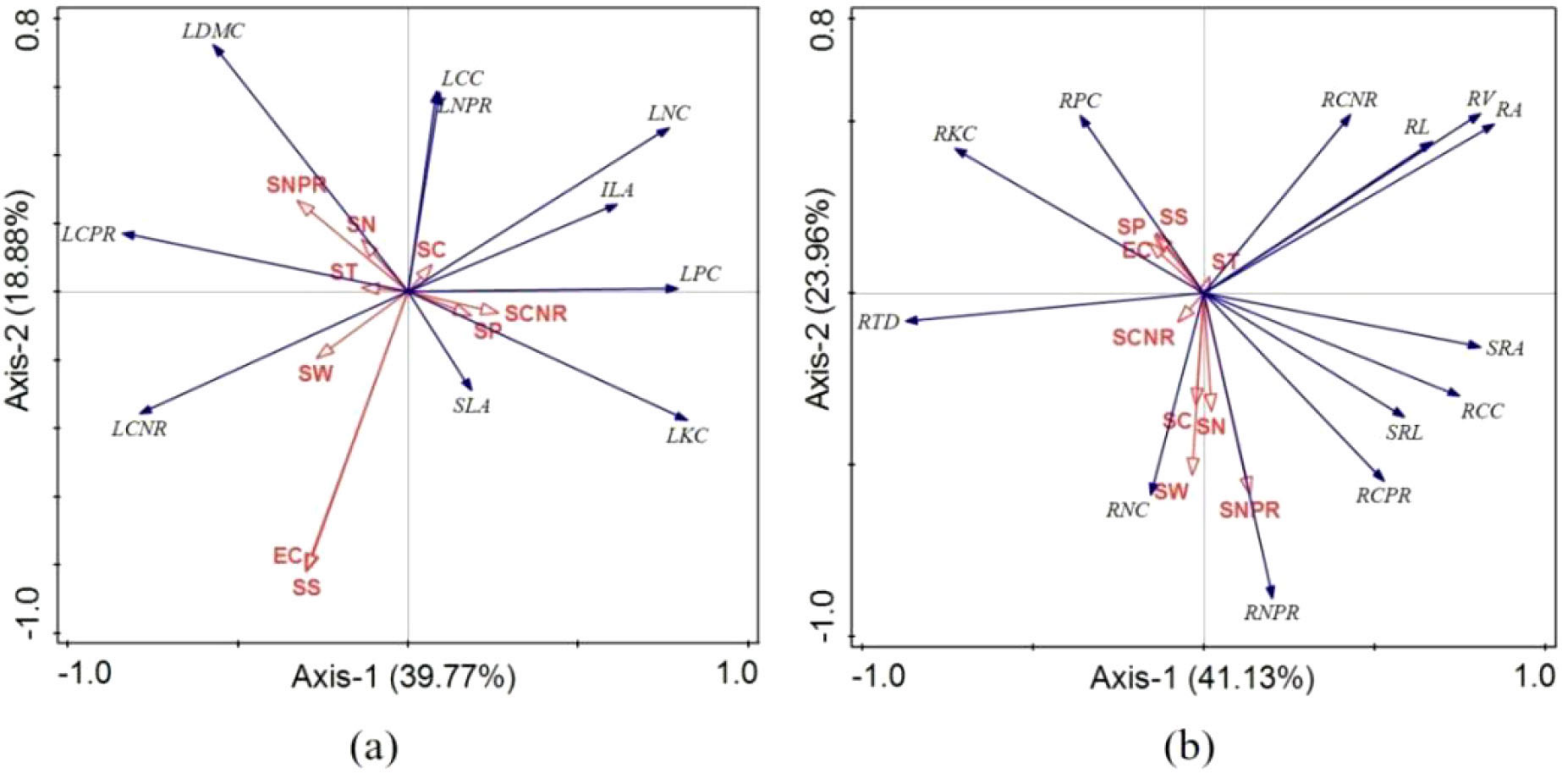
RDA ordination of plant functional traits under mild human disturbance. **(a)** Leaf traits. **(b)** Root traits. SW, soil moisture; ST, soil temperature; SS, soil salinity; EC, soil conductivity; SN, soil total nitrogen; SP, soil total phosphorus; SC, total soil organic carbon; SCPR, soil carbon-phosphorus ratio; SCNR, soil carbon-nitrogen ratio; ILA, leaf area; SLA, specific leaf area; LDMC, leaf dry matter content; LCC, leaf carbon content; LNC, leaf nitrogen content; LPC, leaf phosphorus content; LKC, leaf potassium content; LCNR, leaf carbon-nitrogen ratio; LCPR, leaf carbon-phosphorus ratio; LNPR, leaf nitrogen-phosphorus ratio; RL, root length; RV, root volume; RA, root surface area; SRL, specific root length; RTD, root tissue density; SRA, specific root area; RCC, root carbon content; RNC, root nitrogen content; RPC, root phosphorus content; RKC, root potassium content; RCNR, root carbon-nitrogen ratio; RCPR, root carbon-phosphorus ratio; RNPR, root nitrogen-phosphorus ratio.

RDA analysis identified varying environmental drivers of plant traits across disturbance intensities (**Figure 7**). In lightly disturbed wetlands (**Figure 7a**), leaf traits were primarily influenced by EC and SS (19.9% each), showing strong negative correlations with LCC and LNPR. Root traits (**Figure 7b**) were mainly affected by SW (12.4%) and SCNR (11.1%), with SW positively correlated with RNC and negatively with RCNR.

**Figure 8:**
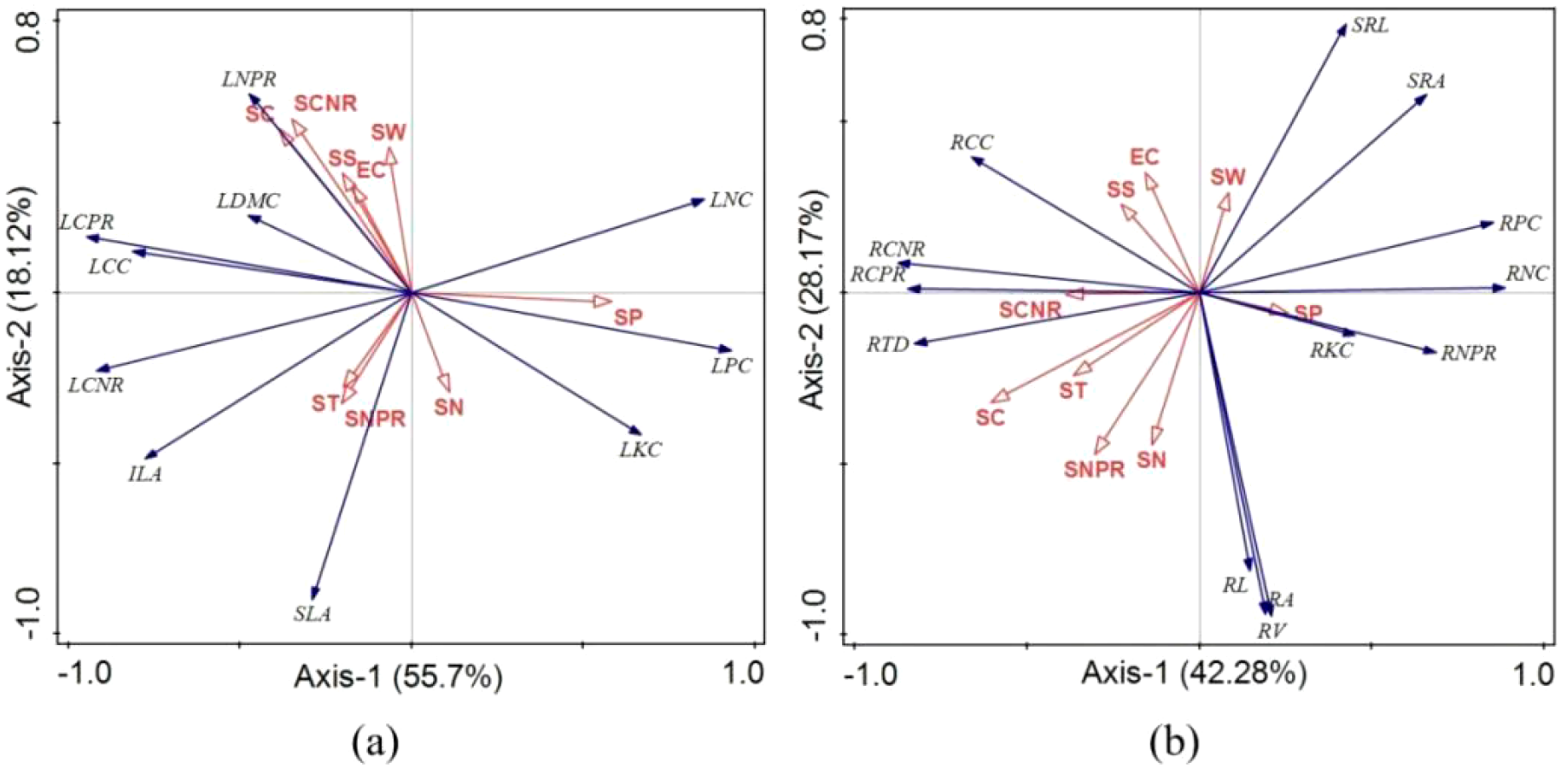
RDA ordination of plant functional traits under mild human disturbance. **(a)** Leaf traits. **(b)** Root traits. Same as **Figure 8**.

In moderately disturbed wetlands (**Figure 8a**), SP emerged as the dominant factor for leaf traits (22.5%), exhibiting strong positive correlation with LPC and negative correlations with LCPR and LCC. For root traits (**Figure 8b**), SC was the primary driver (22.9%), showing positive correlation with RTD and negative correlations with SRA and RPC.

**Figure 9:**
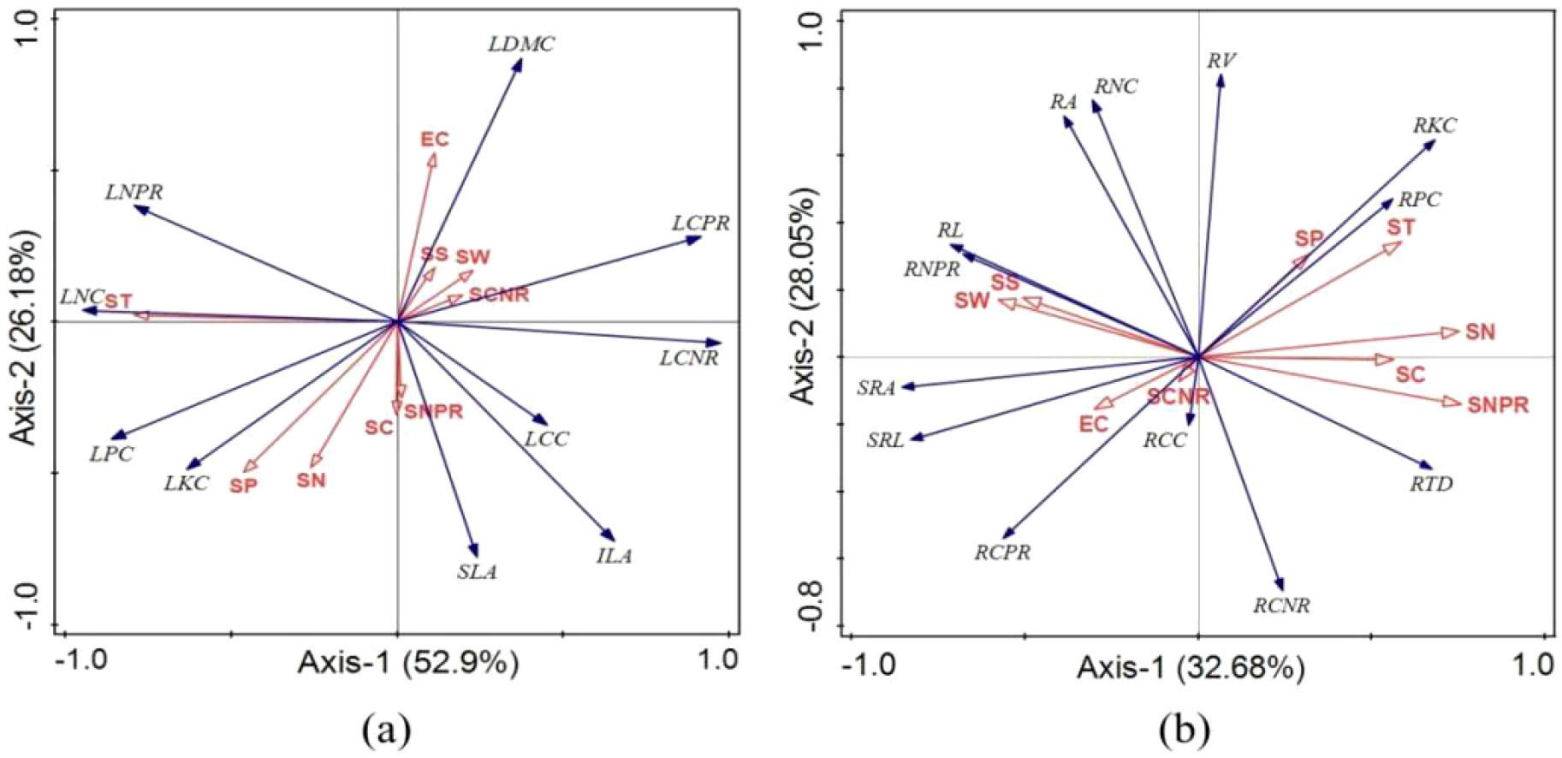
RDA ordination of plant functional traits under mild human disturbance. **(a)** Leaf traits. **(b)** Root traits. Same as **Figure 8**.

Under severe disturbance (**Figure 9a**), ST (36.6%) and SP (22.6%) were the main factors affecting leaf traits. ST positively correlated with LNC and negatively with LCNR, while SP positively correlated with LKC and negatively with LDMC. For root traits (**Figure 9b**), SN (24.1%) and SP (23.2%) were dominant, with SN negatively correlated with SRA and SRL, and SP positively correlated with RPC and RKC but negatively with RCPR.

## 4 Discussion

### 4.1 Trait variations across disturbance gradients

Our study demonstrates systematic variations in plant functional traits along the human disturbance gradient in the Sanjiang Plain wetlands. The observed patterns reflect distinct plant adaptive strategies to changing environmental conditions.

#### 4.1.1 Leaf trait responses

The elevated LA and LDMC under mild disturbance indicate investment in durable leaf structures with longer lifespans (**Figure 2**), consistent with the conservative resource-use strategy typically associated with stable, nutrient-rich environments. In contrast, the significantly higher SLA under severe disturbance reflects a shift toward rapid resource acquisition, enabling plants to capitalize on temporarily available resources in disturbed habitats. This strategic shift along the leaf economics spectrum represents a fundamental trade-off between persistence and rapid growth.

The stoichiometric patterns further elucidate these adaptive mechanisms (**Figure 3**). The higher LCC under mild disturbance supports structural investment, while the elevated LNC and LPC under moderate disturbance suggest efficient nutrient retention in less fertile conditions. The distinct LNPR and LCPR patterns across disturbance levels indicate varying nutrient limitation regimes, with phosphorus limitation becoming increasingly pronounced in less disturbed systems.

#### 4.1.2 Root trait plasticity

Root morphological traits exhibited remarkable plasticity across disturbance gradients (**Figure 4**). The developed root systems with higher RV and RA under mild disturbance represent substantial carbon investment in belowground exploration. Conversely, the elevated SRL under moderate and severe disturbance reflects a shift toward efficient soil exploration with minimal carbon investment—a classic response to nutrient stress.

The stability of root stoichiometric traits compared to leaf traits (**Figure 5**) suggests stronger homeostasis in belowground organs. However, the significantly higher RCC and RCPR under mild disturbance indicate greater carbon allocation to root structures in undisturbed conditions, supporting the observed morphological differences.

### 4.2 Environmental drivers and adaptive mechanisms

The correlation analyses reveal how soil factors shape trait expression (**Figure 6**). The positive relationships between SW and acquisitive root traits (SRL, SRA) demonstrate how water availability drives root economic strategies. Similarly, the negative correlation between soil nutrients and SRL supports the resource conservation theory—in nutrient-rich soils, plants reduce investment in nutrient-acquisition structures.

The RDA results further elucidate the changing importance of environmental drivers across disturbance levels (**Figures 7**-**9**). In mildly disturbed wetlands, where soil factors explain less trait variation (58.65-65.09%), plant traits appear influenced by biotic interactions and historical contingencies. However, as disturbance intensifies, soil factors become dominant drivers, explaining 73.82-79.08% of trait variation in severely disturbed wetlands.

### 4.3 Integrated response strategies

Our findings reveal a continuum of plant strategies across the disturbance gradient. In mildly disturbed wetlands, plants exhibit conservative traits with high tissue density and carbon content, representing a “slow-return” investment strategy. Moderately disturbed environments select for balanced resource allocation with efficient nutrient retention. Under severe disturbance, plants shift toward acquisitive traits with high SLA and SRL, adopting a “fast-return” strategy to capitalize on dynamic conditions.

These patterns align with the leaf and root economic spectra frameworks but extend them by demonstrating how multiple traits coordinate across organs in response to complex environmental gradients. The Sanjiang Plain wetlands thus provide a model system for understanding how human disturbance reshapes plant adaptive strategies through environmental filtering.

## 5 Conclusion

This study demonstrates that wetland herbaceous plants in the Sanjiang Plain exhibit distinct functional trait adjustments and ecological strategies along anthropogenic disturbance gradients. Under mild disturbance, plants developed robust leaf structures and root systems adapted to nutrient-rich conditions, though leaf growth remained susceptible to phosphorus limitation. As disturbance intensified to moderate levels, plants increased nitrogen and phosphorus content to cope with nutrient-poor soils while developing finer root systems to enhance nutrient acquisition efficiency. Under severe disturbance, plants displayed intermediate leaf traits but the most pronounced root responses, with specific root length showing particular sensitivity to soil water and salt content. These trait variations reflect a strategic shift along the resource economics spectrum: plants in moderately disturbed wetlands adopted a “slow investment-return” strategy characterized by resource conservation, while those in heavily disturbed wetlands shifted toward a “rapid investment-return” strategy prioritizing fast resource acquisition. These findings collectively reveal how wetland plants adjust their functional traits to adapt to varying environmental conditions under human disturbance, providing important insights for wetland ecosystem conservation and restoration.

## Data Availability

The original contributions presented in the study are included in the article/supplementary material. Further inquiries can be directed to the corresponding author.
