# Differences in functional traits of herbaceous plants in Sanjiang plain wetland under human disturbance gradient and their response strategies to environmental changes

**DOI:** 10.3389/fpls.2026.1797278

**Published:** 2026-02-12

**Authors:** Qiuyu Meng, Zihe Liu, Naixu Guo, Jiping Liu

**Affiliations:** 1 College of Geographic Science and Tourism, Jilin Normal University, Siping, China; 2 College of Life Sciences, Jilin Normal University, Siping, China

**Keywords:** environmental factors, human interference gradient, plant functional traits, response strategy, Sanjiang Plain

In the published article, there was a mistake in the **Funding** statement:

The author(s) declared that financial support was not received for this work and/or its publication.

The correct statement should be “The author(s) declared that financial support was received for this work and/or its publication. This research work was funded by the National Natural Science Foundation of China (Project Number: 42271125)”.

